# A successful prediction of the record CO_2_ rise associated with the 2015/2016 El Niño

**DOI:** 10.1098/rstb.2017.0301

**Published:** 2018-10-08

**Authors:** Richard A. Betts, Chris D. Jones, Jeff. R. Knight, Ralph. F. Keeling, John. J. Kennedy, Andrew J. Wiltshire, Robbie M. Andrew, Luiz E. O. C. Aragão

**Affiliations:** 1 Met Office Hadley Centre, Exeter, UK; 2 Global Systems Institute, University of Exeter, Exeter EX4 4QE, UK; 3 Scripps Institution of Oceanography, University of California San Diego, La Jolla, CA, USA; 4 CICERO Center for International Climate Research, Oslo, Norway; 5 National Institute for Space Research, Remote Sensing Division, Av. Dos Astronautas 1758, Jardim da Granja, São José dos Campos 12.227-010, Brazil; 6 College of Life and Environmental Sciences, University of Exeter, Exeter EX4 4QF, UK

**Keywords:** El Niño, carbon dioxide rise, Mauna Loa, seasonal forecast, terrestrial biosphere, emissions

## Abstract

In early 2016, we predicted that the annual rise in carbon dioxide concentration at Mauna Loa would be the largest on record. Our forecast used a statistical relationship between observed and forecast sea surface temperatures in the Niño 3.4 region and the annual CO_2_ rise. Here, we provide a formal verification of that forecast. The observed rise of 3.4 ppm relative to 2015 was within the forecast range of 3.15 ± 0.53 ppm, so the prediction was successful. A global terrestrial biosphere model supports the expectation that the El Niño weakened the tropical land carbon sink. We estimate that the El Niño contributed approximately 25% to the record rise in CO_2_, with 75% due to anthropogenic emissions. The 2015/2016 CO_2_ rise was greater than that following the previous large El Niño in 1997/1998, because anthropogenic emissions had increased. We had also correctly predicted that 2016 would be the first year with monthly mean CO_2_ above 400 ppm all year round. We now estimate that atmospheric CO_2_ at Mauna Loa would have remained above 400 ppm all year round in 2016 even if the El Niño had not occurred, contrary to our previous expectations based on a simple extrapolation of previous trends.

This article is part of a discussion meeting issue ‘The impact of the 2015/2016 El Niño on the terrestrial tropical carbon cycle: patterns, mechanisms and implications’.

## 1. Introduction

By September 2015, indices of the El Niño Southern Oscillation (ENSO) were showing strong El Niño conditions [1], and forecast centres were predicting a further substantial strengthening of the El Niño over the coming months. A large body of previous work had demonstrated a strong correlation between ENSO and short-term fluctuations in the rate of rise of atmospheric CO_2_ concentration, with El Niño events generally followed by a larger annual rise in atmospheric CO_2_ concentration [2–6], except after large volcanic eruptions [7]. Another point of interest at that time was that the annual cycle in CO_2_ concentrations had been varying around the symbolic threshold of 400 ppm for the previous 2 years, and in September and October 2015, the annual minimum monthly mean concentration measured at Mauna Loa was 397.5 ppm and 398.28 ppm, respectively [8]. In October 2015, Keeling [9] noted that extrapolation based on previous trends would indicate that the following year would still see annual minimum concentrations below 400 ppm, but that the forecast large El Niño would be expected to lead to a faster CO_2_ rise causing concentrations to remain above 400 ppm for all of 2016. Therefore, Keeling informally predicted that October 2015 would be the last time that monthly concentrations below 400 ppm would be seen in the Mauna Loa record.

**Figure 1.**
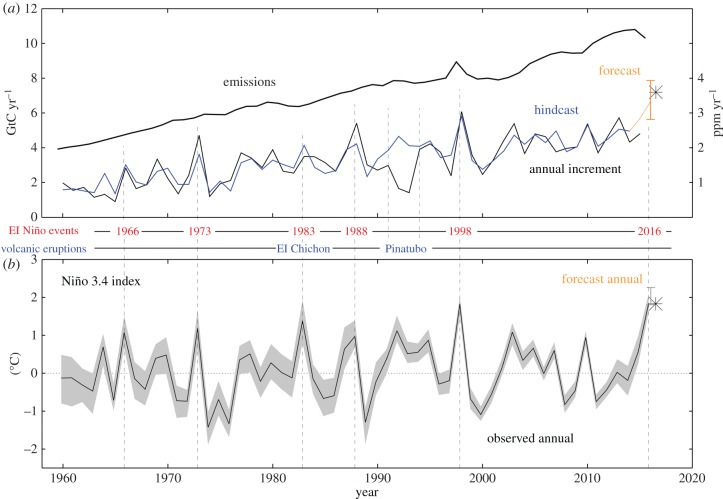
Identifying, testing, forecasting and verifying the relationship between Niño 3.4 SST anomalies and Mauna Loa CO_2_ annual increments. (*a*) Anthropogenic CO_2_ emissions (thick black); CO_2_ annual increments from observations (thin black), reconstructed from regression against emissions and Niño 3.4 anomaly before 2015 (blue) and forecast for 2016 using the forecast annual mean SST (orange). The black star shows the observed CO_2_ annual increment. (*b*) Annual (April to March) mean sea surface temperature anomalies in the Niño 3.4 region from HadSST3 ensemble of homogenized observations (grey) and its median (black line), with the forecast final annual mean from HadSST3 observations from 1 April to 31 October combined with GloSea5 forecast SSTs for 1 November 2015 to 30 March 2016 (orange). The black stars show the observed annual SST anomaly and annual CO_2_ increments. Also shown are the years of major El Niño events (red text), and major volcanic eruptions (blue text) when the relationship between the Niño 3.4 SST and CO_2_ annual increment breaks down due to the cooling effect of volcanic aerosols. The forecast method cannot account for the effects of major volcanic eruptions occurring after the forecast has been issued, due to their unpredictable nature.

Following this, Betts *et al.* [10] published a formal prediction of the annual mean rise in CO_2_ concentration between 2015 and 2016, using observed and forecast sea surface temperatures (SSTs) in the equatorial east Pacific Ocean and a statistical relationship between SSTs and the annual CO_2_ increment. Our published forecast was for the annual mean CO_2_ concentration for 2016 measured at Mauna Loa to be 3.15 ± 0.53 ppm higher than that for 2015 (figure 1). This was larger than any annual increment in the Mauna Loa record so far, including 1997–1998 following the previous very large El Niño. (It is important to note that, as in our previous work [5,6], the focus here is on annual increments—the difference between annual means for successive calendar years—as opposed to annual growth rates which are the rates of change across a calendar year, as used in other studies such as the Global Carbon Budget [11–13].) From this forecast annual increment, we further forecast that the annual mean Mauna Loa concentration for 2016 would be 404.45 ± 0.53 ppm, with monthly values varying between a maximum of 407.57 ± 0.53 ppm in May and a minimum of 401.48 ± 0.53 ppm in September. Therefore this formal published forecast [10] supported the informal prediction by Keeling [9] that CO_2_ at Mauna Loa would remain above 400 ppm throughout 2016 as a result of the El Niño.

Here, we provide a formal verification of the Betts *et al.* [10] forecast against observations, including verification of individual components of the forecast. We discuss the processes involved in the impact of the El Niño on the CO_2_ rise and highlight some key issues such as precise definitions of terms and quantities which are important to note in future forecasts. We also apply the same methodology to the observed values of emissions and SSTs to estimate the contribution of the El Niño to the CO_2_ rise between 2015 and 2016, and assess whether the CO_2_ concentration would have remained below the symbolic 400 ppm threshold all year round in the absence of the El Niño.

## 2. Details of the forecast process

The CO_2_ forecast used a multiple linear regression of annual CO_2_ increments (ΔCO_2_) against annual anthropogenic emissions (*ɛ*) and the annual mean anomaly in sea surface temperatures in the region of the equatorial Pacific Ocean characterizing ENSO activity (*N*):2.1
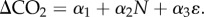
This had previously been used to explore relationships between ENSO and the variability in CO_2_ rise [6], using SST anomalies averaged over the Niño 3 region of the equatorial Pacific (5° N–5° S, 150° W–90° W). For the 2016 CO_2_ forecast, the regression was recalculated using SSTs averaged over the Niño 3.4 region (5° N–5° S, 170° W–120° W) because the El Niño conditions were focused more in the eastern Pacific rather than the central Pacific as in 1997–1998. Previous work [6] had shown that when this regression was used to reconstruct CO_2_ increments between successive years *i* − 1 and *i*, the strongest correlation with observations was seen when *N* was the annual mean from 1st April in year *i* − 1 to 31st March in year *i*, so this period was used here. Annual mean April–March SSTs were taken from the HadSST3.1.1.0 dataset [14,15] and anomalies in °C calculated relative to the 1961–1990 mean. The recalculated regression used the Global Carbon Project dataset of emissions up to 2014 [11]—emissions data are published for calendar years, so the regression used the annual total emissions for years *i* − 1, expressed in GtC. CO_2_ concentrations were taken from the Mauna Loa dataset maintained by the Scripps Institution of Oceanography [8]—annual means over the calendar year were derived by averaging the published monthly mean CO_2_ concentrations. The Mauna Loa measurements were chosen as the focus of the forecast as they provide a very specific, precisely measured quantity—in contrast, the global mean concentration relies on estimates and assumptions, which introduce additional uncertainties. Although CO_2_ is measured at other sites, Mauna Loa is the original measurement site and provides the longest record as well as being of historical interest.

Using the above datasets to calculate the multiple linear regression over the period 1959–2014, using the data available in October 2015, resulted in the following values of the regression coefficients: *α*_1_ = −0.132 ppm yr^−1^ ; *α*_2_ = 0.415 ppm yr^−1^ °C^−1^ ; *α*_3_ = 0.237 ppm GtC^−1^.

The forecast of the annual CO_2_ increment between 2015 and 2016 was calculated at the end of 2015 using equation (2.1) and the above values of the regression coefficients. Since observational data on global emissions for 2015 were not yet available at that time, *ε* was a projection of emissions for 2015 [11] (table 1). Observed SSTs were available up to October 2015, so SSTs from the Met Office seasonal forecast model GloSea5 [16] were used for the remainder of the required period up to 31 March 2016 and the annual mean taken of the observed and forecast SSTs. Both the observational and forecast data are ensemble products. In the case of the observations, the range of estimates reflects uncertainties arising from the sampling and measurement error of the SST observations used to construct the dataset. The SST predictions are an ensemble to reflect the uncertainty arising from sensitivity to the precise initial state of the simulated ocean and atmosphere. The uncertainty in the annual mean combined observed and forecast SSTs was ±0.2°C (2 s.d., from the ensemble of seasonal forecast simulations).

**Table 1.** Inputs and results for the published 2016 CO_2_ forecast (column 3) and corrected forecast (column 4) compared with observations (column 5). Regression coefficients *α*_1_, *α*_2_ and *α*_3_ (rows 2–4) were used with the annual mean sea surface temperature anomaly *N* (row 5) and annual total global CO_2_ emissions *ɛ* (row 6) in equation (2.1) to forecast the annual CO_2_ increment ΔCO_2_ (row 7) and hence the annual mean CO_2_ concentration (row 8). Column 3 shows the values used in the published forecast [16] which included two mistakes (see §2), and column 4 shows the corrected forecast calculation as it should have been with the information available at the end of 2015. Column 5 shows the observed values. *N* is the mean over the Niño 3.4 region for 1 April 2015 to 31 March 2016; for the published and corrected forecasts, *N* used observed SSTs from HadSST3.1.1.0 from 1 April to 31 October combined with forecast SSTs from the GloSea5 model for 1 November to 31 March. The observed SSTs are from the updated HadSST3.1.1.1 alone. *ɛ* is the total emissions over January to December. The forecast used a projection of *ɛ* published in 2015 [11], and the observed *ɛ* was published in 2016 [12]. The forecast CO_2_ increment and concentrations were subject to an error estimate of ±0.53 ppm.

	period	published forecast	corrected forecast	observed
*α*_1_ (ppm yr^−1^)		−0.132	−0.132	
*α*_2_ (ppm yr^−1^ °C^−1^)		0.415	0.415	
*α*_3_ (ppm GtC^−1^)		0.237	0.237	
*N* (°C)	Apr 2015–Mar 2016	2.02 ± 0.23	2.02 ± 0.23	1.85 ± 0.19
*ɛ* (GtC)	Jan–Dec 2015	10.3	10.84	11.1
ΔCO_2_ (ppm)	2016–2015	3.15	3.28	3.39
CO_2_ (ppm)	annual mean 2016	404.45	404.17	404.28

**Figure 2.**
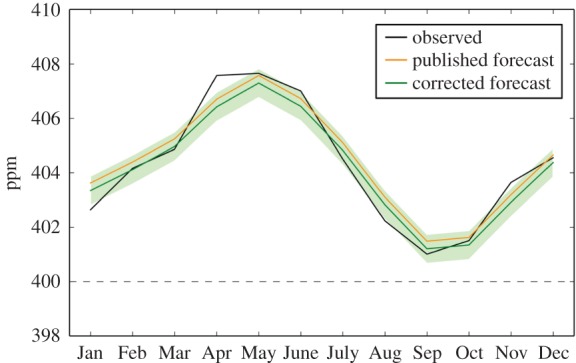
Observed and forecast monthly mean CO_2_ concentration at Mauna Loa for 2016. Black: observations. Orange: values from the published forecast. Green: values from the corrected forecast.

The primary quantity being forecast was the annual increment in CO_2_ concentration (table 1). We then used this to forecast the annual mean CO_2_ concentration at Mauna Loa for 2016 (table 1) by adding the forecast increment to the observed 2015 value. We then added an adjustment factor for the difference between each monthly value and the annual mean (electronic supplementary material, table S1) in order to forecast the monthly mean CO_2_ concentration for each month of 2016 (figure 2; electronic supplementary material, table S2). This relies on the assumption that the mean annual cycle in CO_2_ concentration over previous years would represent the annual cycle in 2016. The monthly mean CO_2_ concentration for September was of particular interest, because this would be the lowest value of the year and a key question was whether this would remain above 400 ppm. We had forecast the mean CO_2_ concentration for September 2016 to be 401.8 ± 0.53 ppm, which suggested that October 2015 had been the last time that monthly mean concentrations below 400 ppm would be seen in the Mauna Loa record.

It should be noted that other studies such as the Global Carbon Project [11–13] quantify the annual CO_2_ rise differently, using the in-year growth rate defined as the change between the start and end of the calendar year, as opposed to the difference between the annual means of consecutive calendar years as used here. Implications of these different approaches are discussed below.

## 3. Verification of the 2016 Mauna Loa CO_2_ forecast

The observed annual mean CO_2_ concentration in 2016 was 404.28 ppm [8], so with the 2015 concentration having been 400.89 ppm [8], the observed annual increment was 3.39 ppm—close to the forecast central value of 3.15 ppm and well within the error estimate of ±0.53 ppm (table 1).

The observed mean 2016 concentration was also close to the published forecast value of 404.45 and again within the error estimate. The observed mean concentration for September 2016 was 401.01 ppm, again within the forecast error estimate (figure 2; electronic supplementary material, table S2). It was therefore confirmed that, as predicted by Keeling [9] and Betts *et al.* [10], October 2015 had indeed been the last instance in the Mauna Loa record with monthly mean CO_2_ concentration below the iconic level of 400 ppm.

A point to note is that the Betts *et al*. [10] published forecast of mean CO_2_ concentration included two mistakes. One arose from a misreading of the calculation of estimated fossil fuel emissions for 2015 published by the Global Carbon Project [11]. The estimated 2015 emissions were incorrectly taken to be 10.3 GtC, when in fact the published estimate was 10.84 GtC. Applying this to equation (2.1), the forecast annual increment should therefore have been 3.28 ppm. This would have been a more accurate forecast of the annual increment than the published value. The second mistake arose from a typographical error when drafting the paper and adding the forecast annual increment to the observed 2015 CO_2_ concentration. Correcting for both these mistakes, with a forecast annual increment being 3.28 ppm and the observed concentration for 2015 being 400.89, the forecast annual mean concentration for 2016 should have been 404.17 ± 0.53 ppm. Again, this would have been a more accurate forecast than the published value. The corrected forecast annual mean concentration was 0.28 ppm lower than the published value, so the forecast monthly values should therefore also have been forecast as 0.28 ppm lower than those in the published forecast. The forecast September concentration should therefore have been 401.20 ± 0.53 ppm. Nevertheless, the impact of these mistakes on the calculations was small in comparison with the impact of the SST anomaly, and both the published and corrected forecast values agreed with observations within the published error ranges.

The error estimates for the monthly concentrations had been given as ±0.53 ppm, the same as for the annual concentration. The observations fell within this range of the corrected forecast values for eight months (figure 2): the forecast overestimated the concentration in January and August, and underestimated it in April and November. The observed April concentration was notably high in comparison with expectations from the mean seasonal cycle, being almost as high as the concentration in May.

In our original forecast, we also noted that recent observations included instances of CO_2_ rise anomalies of up to 0.6 ppm above the annual increment expected from emissions alone, due to climate variability unconnected with ENSO. Therefore, for the El Niño impact to be outside expectations from the normal trend plus non-ENSO variability, the CO_2_ increment needed to be at least 2.7 ppm. This provided a null projection showing that the successful ENSO-based method added value.

**Figure 3.**
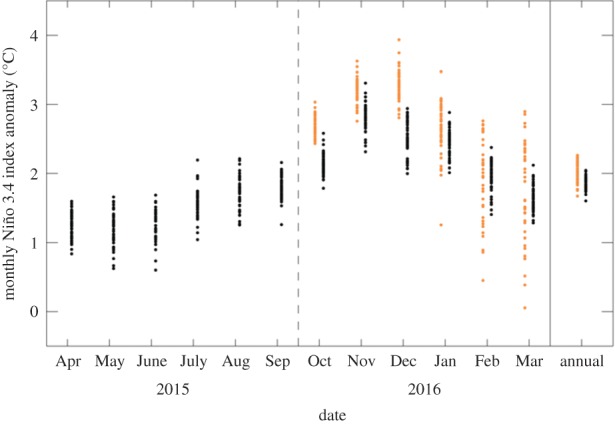
Verification of Niño 3.4 SST inputs to the CO_2_ forecast. The black symbols show the monthly mean observational data (April 2015–March, with those from October 2015 onwards unknown at the time of making the forecast) and the orange symbols the seasonal forecast data (October 2015–March 2016) used in making the CO_2_ forecast. April 2015–March 2016 annual averages are shown on the right, with orange symbols here corresponding to the combination of observed and forecast data used in the 2016 CO_2_ forecast. Each symbol for the observational data represents a single Niño 3.4 index anomaly realization drawn from an ensemble sea surface temperature product, and each orange symbol is a member of an ensemble seasonal forecast.

The Niño 3.4 SST forecast used as input to the CO_2_ rise forecast also verified well against observations (figure 3). Niño 3.4 anomaly values subsequently available show the strong El Niño event peaking in late 2015 and continuing into early 2016. The seasonal predictions also showed the event peaking in late 2015. During the peak period, the forecast tended to be warmer than the observations, although there was always some overlap between the observed and predicted Niño 3.4 distributions. Later in the forecast (in early 2016), the centre of the forecast distribution was better aligned with the observed estimates. The range of estimates of the annual mean Niño 3.4 used in making the 2016 CO_2_ forecast (i.e. the combination of observed and forecast data) showed a very good agreement with the range based on observations for the whole year (which were not available at the time of the forecast). The mean of realizations for the 12-month period was 1.85 ± 0.19°C, slightly less than the predicted value used in making the forecast (2.02 ± 0.23°C). This was not a significant difference considering the strong ensemble overlap and the year-to-year differences in annual mean Niño 3.4, which ranged from approximately −1.5 to +2.0°C (figure 1). As a result, we can conclude that the SST used as input to the CO_2_ prediction was accurately forecast.

Care should be taken in the interpretation of probabilistic forecasts and their verification. The forecast of the CO_2_ concentration is provided as a central estimate and an uncertainty range, with the uncertainty range corresponding to 2 s.d. The original forecast stipulated that a ‘successful’ forecast would fall within this range; however, the forecast is probabilistic, with the central estimate and uncertainty specifying a probability distribution. Therefore, even if the central estimate and uncertainty are correct, there is still a chance that the observed value will lie outside the forecast range (even leaving aside volcanic eruptions which were explicitly excluded). In any year, the probability that the observed value will fall outside the forecast range is around 4%. Assuming that forecast errors are independent and normally distributed then over 5 years, the probability that at least 1 year will fall outside the range is about 21%. Over 10 years, the probability rises to 37%. For 15 years, the probability is about 50 : 50. In the long term, a good forecast would yield a predictable rate of ‘failures’.

## 4. Understanding the success of the forecast and potential improvements

The success of a forecast relies on two main factors: the methodology and the input data. The following discussion reflects on these.

### (a) Process understanding supporting the statistical relationship between El Niño and the CO_2_ rise annual increment

Our successful forecast used a method that was partly process-based (the SST forecast) and partly statistical (the relationship between SSTs and the CO_2_ increment). Does an examination of the carbon cycle processes support the expectations from the statistical relationship?

A large body of previous work has shown that the main contribution to larger CO_2_ growth rates associated with El Niño events is reduced net carbon uptake by the terrestrial biosphere [2–6]. This is slightly offset by increased net uptake of CO_2_ by the oceans due to reduced outgassing because of decreased upwelling of deep water with high carbon content [5]. Here, we estimate the contribution of the terrestrial biosphere to the record rise associated with the 2015/2016 El Niño by using the JULES land surface model [17]. We focus here on net biome productivity (NBP) defined as:4.1
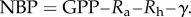
where GPP is Gross Primary Productivity, i.e. uptake of carbon by plant photosynthesis, *R*_a_ is autotrophic respiration, *R*_h_ is heterotrophic respiration and *γ* is the release of carbon through disturbance mechanisms such as anthropogenic land use and fire.

JULES and other process-based terrestrial biosphere models generally simulate negative (or reduced) global NBP in El Niño years [18]. To simulate the response to the 2015/2016 El Niño, we use the current ‘carbon cycle configuration’ (JULES-C1.1) as used in the Global Carbon Budget [13]. We simulate land–atmosphere carbon fluxes over the period from 1860 to 2016, driving JULES-C-1p1 with observed changes in climate [19,20], global mean CO_2_ concentration and land use change [21,22].

To assess the impact of the 2015/2016 El Niño, we first calculate NBP for the period 1 June 2015 to 31 May 2016—this is the 12-month period centred around the peak of the SST anomaly in December. We then calculate the NBP for the same period starting in each of the previous 10 years and find the mean of this as a comparison with 2015–2016.

**Figure 4.**
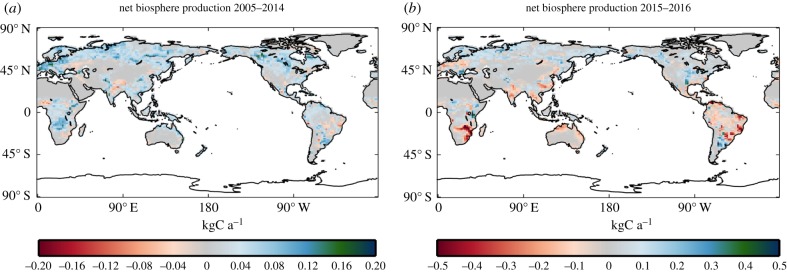
Spatial pattern of annual atmosphere–land carbon fluxes averaged over 2005–2014 (*a*) and for June 2015 to May 2016 (*b*), simulated by the JULES model. Positive values indicate carbon uptake from the atmosphere by the land.

On average over 2005–2014, JULES simulates most of the terrestrial biosphere to be a net sink of carbon in the annual mean (figure 4a). In JULES, this is largely due to CO_2_ fertilization, with some contribution of climate change, particularly warmer regional climates at higher latitudes. Other terrestrial biosphere models produce similar results [23,24]. Some regions show a near-zero or negative NBP due to losses from anthropogenic land use change offsetting or even dominating CO_2_ fertilization and climate effects. Negative NBP in some regions may also be due to regional climate changes causing conditions to be less favourable for vegetation growth or enhancing soil respiration.

**Figure 5.**
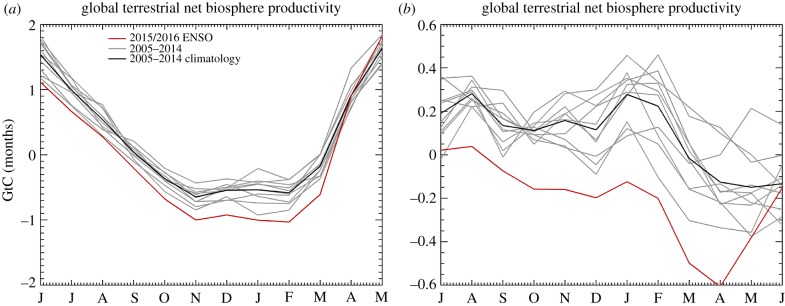
Land–atmosphere carbon fluxes simulated by JULES: global total (*a*) and total over the tropics (±30° of latitude), (*b*). Highlighted is the 2015/2016 El Niño (red) and the 2005–2014 climatology (black) and individual years (grey).

The land–atmosphere carbon fluxes vary across the year. JULES simulates a global peak uptake in June coinciding with the boreal summer and a net carbon loss during the boreal winter (figure 5a). The seasonal cycle in uptake reflects the asymmetry in land mass between the North and Southern Hemispheres. Overall, however, the terrestrial biosphere is simulated to be net sink of carbon over the previous decade (2005–2014) averaging 2.82 GtC yr^−1^.

During the 2015/2016 El Niño, the temperate and boreal regions are simulated to have become weaker sinks of carbon, and much of the tropics became a net source (figure 4b). Substantial areas of Brazil, particularly the Atlantic Forest region, Southern Africa, South and southeast Asia and tropical Australia, become sources of carbon to the atmosphere (figure 4). The transition to source is not consistent across the tropics, with Central Africa just showing a weakening in the sink. Europe also switches from a net sink to a net source.

This weakening of the global land carbon sink is simulated throughout the 12-month period (figure 5b). In June 2015–May 2016, JULES simulates lower global total carbon uptake in comparison with means for each calendar month over the previous decade. The switch from a net source to net sink in the tropics occurs all year (figure 5b). During the period of El Niño, the land becomes a net source of 1.12 GtC, in contrast to the net sink over the previous decade (2005–2014). The net result is an additional 3.94 GtC of CO_2_ in the atmosphere than would have been the case if 2015/2016 had been the same as the previous decade, equivalent to 1.86 ppm.

The regional climate anomalies associated with the 2015/2016 El Niño therefore reduced simulated terrestrial carbon uptake in JULES. Overall, JULES simulated the tropical land areas to have switched from a sink of 1.07 GtC to a source of 2.63 GtC.

The JULES simulations do not explicitly represent wildfire, which can also play a role in carbon emissions from ecosystems during El Niño events. In the 1997/1998 El Niño, extremely high fire emissions from Indonesian forests contributed to the faster CO_2_ rise [25], but Indonesian fire emissions were much smaller in 2015/2016 [26]. In Amazonia, fire is now decoupled from land use—fire activity relates more strongly to climate variability than to direct ignitions related to the deforestation process [27]. The 2015/2016 El Niño appears to have led to more fire in Amazonia, but mainly in 2015. Fire activity in Amazonia was high in 2015, with 19% (799 293 km^2^) of Brazilian Amazonia affected by at least one active fire detection, experiencing significant positive (*p* < 0.1) active fire anomalies [27]. In this same year, the area affected by positive active fire anomalies greater than 2s.d. (*p* < 0.05–628 901 km^2^) was approximately twice as large as anomalies observed during the 2005 and 2010 droughts (363 245 and 388 803 km^2^, respectively). It was estimated that gross committed CO_2_ emissions from Amazonia for 2015 reached a total 0.52 Pg CO_2_ [27]. However, fire emissions from South America are unlikely to have a direct contribution to the 2016 rapid rise in atmospheric CO_2_ as measured at Mauna Loa. Peak fire activity in South America occurs in August and September, so the El Niño impacts on fire emissions from Amazonia are likely to have affected the CO_2_ rise in 2015 rather than 2016.

These model results support previous expectations that the 2015/2016 El Niño caused a weakening of the terrestrial carbon sink, which resulted in the atmospheric CO_2_ concentration rising faster than usual. This suggests that the statistical relationship between Niño 3.4 SSTs and CO_2_ annual increment is supported by understanding of the Earth system processes and appropriate for use in forecasting the CO_2_ rise.

### (b) Shape of seasonal cycle and assumption of stationarity

The shape of the seasonal cycle is assumed to be stationary in our method. This implicitly assumes no change in the growth rate across the year, which is contradicted by of the observations of varying in-year growth rates. Nevertheless, it appears that the assumption of stationarity may be valid for predicting the annual maximum and minimum monthly values, as there is little long-term trend in the amplitude of the seasonal cycle (electronic supplementary material, figure S1).

Regarding the in-year growth rates, 2015 had a large growth rate because the concentrations at the end of the year were anomalously high. Nevertheless, the in-year growth rate was still large across 2016 despite concentrations already being anomalously high at the start of the year. The JULES simulations suggest that the land–atmosphere carbon fluxes were impacted by El Niño until at least June 2016 (figure 5), so it seems that the El Niño impacts on tropical ecosystems lasted longer than the Niño 3.4 SST anomaly which ended in May.

These issues are important in the context of the annual Global Carbon Budget (GCB) calculations [11–13]. That routinely quotes the in-year growth rate, but for the 2017 GCB [13], we provided a forecast which was based on our definition of annual increment [28]. Our forecast of the 2016–2017 annual increment was used to provide CO_2_ concentrations for October–December, which were combined with observed monthly concentrations to give an estimate of the mean CO_2_ concentration for 2017 in time for publication before the end of the year. That combined observed/forecast estimate verified better than the forecast issued at the start of the year [28] when compared with the observed annual increment. There are additional difficulties in forecasting the in-year growth rate a year in advance, because effects of ENSO could affect the end-of-year CO_2_ concentrations but cannot currently be forecast with skill more than a few months in advance. However, the annual GCB calculations are routinely made in or around August, which is when signals of ENSO start to emerge. Therefore, it may be possible to develop a method for forecasting the monthly CO_2_ concentrations for the final few months of the year at this time, for inclusion in annual GCB calculations.

Monthly CO_2_ concentrations measured at a single site such as Mauna Loa may be affected by other factors in addition to those arising from the impacts of climate variability on surface–atmosphere carbon fluxes and the global mean CO_2_ concentration. For example, local wind directions different from the climatological average could make a difference. In such cases, the monthly anomalies would not be representative of the global anomaly—the Mauna Loa concentration is normally regarded as a proxy for the annual global mean concentration, but this may not always be the case, especially on shorter timescales. Investigation of this is outside the scope of the current paper, but could involve comparison with monthly anomalies at other CO_2_ measuring stations around the world. In future Mauna Loa CO_2_ forecasts, it may be appropriate to calculate error estimates for forecast monthly concentration that account for these additional uncertainties, rather than using the same error estimate as for the annual mean forecast.

### (c) Is the forecast robust to the input data? Contribution of input data and regression coefficients to forecast accuracy

The inputs to our CO_2_ forecast themselves include predictions—the SSTs for the coming months and the emissions. We can assess the importance of these by performing a hindcast, i.e. recalculating the forecast using equation (2.1) with input data from observations (table 2). (Note that this is different from the corrected forecast discussed above, which dealt with mistakes made in the forecast production but still only used input data that were available at the time.) Here, we assess whether the retrospective use of actual SSTs and emissions allows such a hindcast to give an improved calculation of the CO_2_ rise compared with the forecast.

**Table 2.** Impact of using observed SST and emissions data on calculation of 2015/2016 CO_2_ annual increment.

	period	corrected forecast	hindcast	observed
*α*_1_ (ppm yr^−1^)		−0.132	−0.132	
*α*_2_ (ppm yr^−1^ °C^−1^)		0.415	0.415	
*α*_3_ (ppm GtC^−1^)		0.237	0.237	
*N* (°C)	Apr 2015–Mar 2016	2.02 ± 0.23	1.85 ± 0.19	
*ɛ* (GtC)	Jan–Dec 2015	10.84	11.1	
ΔCO_2_ (ppm)	2016–2015	3.28	3.27	3.39
CO_2_ (ppm)	annual mean 2016	404.17	404.16	404.28

As noted above, the observed value of *N* was 1.85 ± 0.19°C, slightly smaller than forecast. With a value of *α*_2_ of 0.415 ppm yr^−1^ °C^−1^, this meant that the contribution of the second term of equation (2.1) to the forecast was 0.77 ppm yr^−1^ instead of 0.83 ppm yr^−1^ using the forecast *N*. The contribution of that term was therefore 0.06 ppm smaller than in the forecast. However, the annual mean anthropogenic emissions published for 2015 were larger than the projected value used in the forecast, at 11.1 GtC as opposed to 10.84 GtC. With *α*_3_ = 0.237, the third term of equation (2.1) contributed 2.63 ppm instead of 2.57, an increase of 0.06. The error in the emissions estimate therefore made a smaller contribution than the SST forecast to the error in the CO_2_ annual increment, but partly offset it.

If the forecast had used those numbers, the predicted CO_2_ annual increment would therefore have been 3.27 ppm—slightly further from the observed value of 3.39 ppm, but negligibly so.

Moreover, each year the historical dataset of global emissions is revised based on new information, not only for the most recent year but also previous years (electronic supplementary material, figure S2). This would affect the regression of CO_2_ concentrations against emissions, resulting in revised regression coefficients. We can assess the impact of this by recalculating the forecast using equation (2.1) and revised regression coefficients derived using more recent data (table 3).

**Table 3.** Updated hindcast using regression coefficients for equation (2.1) calculated using different releases of the Global Carbon Budget (GCB) emissions dataset, which present revised historical values and updated Mauna Loa CO_2_ concentrations up to the most recent year. Column 3 shows the coefficients as used in the 2016 CO_2_ forecast [10], derived using data from GCB 2015 [11]. Columns 4 and 5 show the coefficients recalculated with updated emissions and CO_2_ concentration from GCB 2016 [12] and GCB 2017 [13], respectively, and the subsequently recalculated ΔCO_2_ and CO_2_ concentration for 2015–2016.

	period	GCB 2015	GCB 2016	GCB 2017	observed
*α*_1_ (ppm yr^−1^)		−0.132	−0.080	0.045	
*α*_2_ (ppm yr^−1^ °C^−1^)		0.415	0.419	0.426	
*α*_3_ (ppm GtC^−1^)		0.237	0.229	0.214	
*N* (°C)	Apr 2015–Mar 2016	1.85 ± 0.19	1.85 ± 0.19	1.85 ± 0.19	
*ɛ* (GtC)	Jan–Dec 2015	11.1	11.1	11.1	
ΔCO_2_ (ppm)	2016–2015	3.27	3.24	3.21	3.39
CO_2_ (ppm)	annual mean 2016	404.16	404.13	404.10	404.28

Using updated regression coefficients from the GCB 2017 dataset [13] and the same observed input data as above reduced the forecast CO_2_ increment to 3.21 ppm, further from the observed value, but with the forecast error estimate still encompassing the observed value.

The overall conclusion is that neither perfect knowledge of the SSTs and emissions nor updating the regression coefficients on the basis of more recent data would have given a more ‘accurate’ forecast in terms of agreement of the central estimate with observations. However, these updates would also have not caused the forecast to be ‘inaccurate’ in terms of the observations falling outside of the forecast error bars. The forecast using information available at time therefore appears to be robust to the uncertainties in the input information.

## 5. Further analysis: attribution of causes of the observed CO_2_ annual increment

### (a) Comparison with previous CO_2_ rise: mean and previous record

The observed mean CO_2_ rise for the decade prior to 2015 was steady at approximately 2.1 ppm yr^−1^, so the rise of 3.39 ppm in 1 year was a substantial increase. Before 2015, the growth rate did not rise despite an increase in anthropogenic emissions, and this has previously been attributed increased net uptake of carbon by the terrestrial biosphere due to increased CO_2_ fertilization accompanied by a lack of increase in respiration resulting from the temporary slowdown in the rate of global warming [29]. We note that our reconstruction of CO_2_ increments using equation (2.1) captures the hiatus in the rate of CO_2_ rise between ca. 2003 and 2014 (figure 1). Since the only climate-related term in equation (2.1) is the Niño 3.4 SST anomaly, this suggests that the relatively cool conditions in the equatorial Pacific in several of these years may have played a role in the hiatus in the CO_2_ rise. La Niña conditions are associated with smaller annual CO_2_ rises (figure 1), with generally wetter and cooler conditions in many areas. This may be consistent with an emerging understanding of the role of Pacific decadal variability in the global warming hiatus [30–32].

Therefore, although the large increase in CO_2_ rise in 2015/2016 was largely associated with the El Niño, there was also probably a contribution from the cessation of the anomalously slow rate of rise associated with cooler conditions in the tropical Pacific.

The previous largest annual increment, in terms of the difference between annual mean for successive calendar years, was 2.9 ppm between 1997 and 1998 [8] following the large El Niño which occurred in those 2 years. The observed CO_2_ annual increment in 2015/2016 was therefore substantially larger than that following the previous large El Niño event. By contrast, the in-year growth rates for 1998 and 2016 are more similar—this appears to be because the rapid growth associated with the 1997/1998 El Niño occurred mainly across a single year, whereas that in 2015/2016 continued across both years.

The contrasting definitions of year-to-year annual increment and in-year growth rate make an important difference to the year identified with a record growth rate due to the 2015/2016 El Niño. Focusing on the increment in annual mean concentrations leads to 2016 being identified as the year with the most rapid increase in CO_2_ concentrations so far. However, if the in-year growth rate is used, this was larger in 2015 than 2016; the El Niño was becoming strong by September 2015, so impacts on land–atmosphere carbon fluxes and hence CO_2_ concentrations would already have been happening. This is supported by simulations with a land surface model (see §4a).

### (b) Attribution of causes of the record annual CO_2_ rise in 2016 and comparison with 1998

The contribution of the El Niño to the 2015/2016 record CO_2_ rise can be estimated by recalculating equation (2.1) with no SST anomaly, i.e. *N* = 0. Using the observed 2015 emissions of 11.1 GtC and the most recent regression coefficients (column 5 of table 3), we estimate that the 2016 annual CO_2_ increment without El Niño would have been 2.42 ppm. The reconstructed CO_2_ increment with El Niño calculated consistently with this was 3.21 ppm (table 4), so this method suggests that the El Niño increased the 2016 annual CO_2_ increment by 0.79 ppm. We therefore estimate that the El Niño contributed approximately 25% to the record annual CO_2_ rise between 2015 and 2016, with the other 75% being due to anthropogenic CO_2_ emissions.

**Table 4.** Estimating the contribution of El Niño to the annual CO_2_ increment in 2015/2016.

	period	with El Niño	no El Niño	observed
*α*_1_ (ppm yr^−1^)		0.045	0.045	
*α*_2_ (ppm yr^−1^ °C^−1^)		0.426	0.426	
*α*_3_ (ppm GtC^−1^)		0.214	0.214	
*N* (°C)	Apr 2015–Mar 2016	1.85 ± 0.19	0	
*ɛ* (GtC)	Jan–Dec 2015	11.1	11.1	
ΔCO_2_ (ppm)	2016–2015	3.21	2.42	3.39
CO_2_ (ppm)	annual mean 2016	404.10	403.31	404.28

The relative contributions of El Niño and emissions to the 1997/1998 annual mean CO_2_ increment can also be estimated in this way. The Niño 3.4 index for the 1997/1998 El Niño was 1.81°C [14,15], slightly smaller than for 2015/2016, although the 1997/1998 El Niño was different in character to that of 2015/2016 with the temperature anomaly being focused more in the central Pacific. The annual emissions in 1997 were 8.4 GtC [13], including both fossil fuel emissions and land use change. Applying these numbers to the regression gives a hindcast of 2.6 ppm for the CO_2_ increment (table 3), an underestimate compared to the observed rise. This underestimate might be due to the Niño 3.4 index being less representative of the magnitude of the 1997/1998 El Niño; the Niño 3 region might be more representative. The annual CO_2_ increment in the absence of El Niño can then, in principle, be estimated with the same method as applied to the 2015/2016 event above. This results in an estimated ‘no El Niño’ CO_2_ rise of 1.84 ppm and an El Niño contribution of 0.77 ppm. This is similar to that for 2015/2016 (table 5) in terms of the absolute contribution, but is a larger proportion (30%) of the overall rise that year.

**Table 5.** Estimating the contribution of El Niño to the annual CO_2_ increment in 1997/1998.

	period	with El Niño	no El Niño	observed
*α*_1_ (ppm yr^−1^)		0.045	0.045	
*α*_2_ (ppm yr^−1^ °C^−1^)		0.426	0.426	
*α*_3_ (ppm GtC^−1^)		0.214	0.214	
*N* (°C)	Apr 1997–Mar 1998	1.81	0	
*ɛ* (GtC)	Jan–Dec 1997	8.4	8.4	
ΔCO_2_ (ppm)	1997–1998	2.61	1.84	2.9

Three caveats should be borne in mind concerning this. The first is the 10% underestimate of the observed CO_2_ increment. The second is that the Niño 3.4 SST Index may not be as appropriate as Niño 3 for 1997/1998, with the latter probably resulting in a larger estimate of the impact of El Niño. The third is that the emissions data show a clear spike in 1997/1998 (figure 1*a*), and this arises from a spike in the land use emissions [13] associated with major wildfires in tropical peatlands in Indonesia [20]. This temporary increase in land use emissions is therefore being classified as an anthropogenic term. However, it could also be viewed as part of the response to El Niño. Although the fires were ignited by human activity, mainly forest clearance, their extent and impact was magnified substantially by the drought conditions associated with the El Niño.

Despite these caveats, it appears that the main reason why the CO_2_ annual increment in 2015–2016 was larger than 1997–1998 is that anthropogenic emissions increased substantially in that period. Emissions had risen by 2.7 GtC between 1997 and 2015 (an increase of over 30%).

### (c) Would Mauna Loa CO_2_ have remained above 400 ppm all year round without the El Niño?

**Figure 6.**
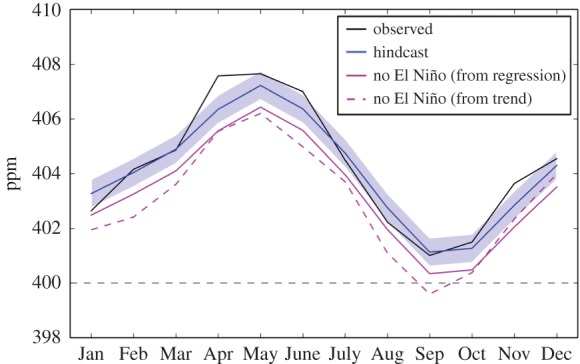
Impact of El Niño on monthly CO_2_ concentrations in 2016. Concentrations including the influence from El Niño are shown with observations (black). The hindcast concentrations (blue) were calculated using observed emissions and observed SSTs that included the El Niño influence (table 4). Concentrations without the influence of El Niño were estimated with two methods: (i) adding the previous decade's trend of 2.1 ppm to the observed monthly concentrations for 2015 (dashed magenta) and (ii) adding the annual increment calculated with a zero El Niño SST anomaly to the 2015 annual mean concentration (table 4), and then adding the same monthly adjustment factors used in the forecast and hindcast (electronic supplementary material, table S1) (solid magenta).

The monthly CO_2_ concentration without the influence of the El Niño can be estimated in two ways. The first method is a simple linear extrapolation of the previous trend of 2.1 ppm yr^−1^, as in our forecast paper [10]. The CO_2_ concentration for September 2015 was 397.50 ppm, so this would give a September mean CO_2_ concentration of 399.60 ppm (figure 6). However, this assumes that the previous trend was representative of the current trend and not itself anomalous—but as noted above, other work [29] suggested that actually the recent trend has been smaller than it should have been, due to the temporary slowdown in the short-term rate of global warming. Therefore, the extrapolation of that trend may not be an appropriate method for estimating the non-El Niño concentration. The second method involves taking the estimated annual ‘no El Niño’ CO_2_ concentration for 2016 (403.31 ppm—table 4) and adding the monthly adjustment factor for September (−2.97 ppm). This gives a value of 400.34 ppm for September 2016 (figure 6). This estimate implies that the monthly mean CO_2_ could have remained above 400 ppm all year round in 2016 even without the El Niño.

The second method relies on the assumption of the stationary amplitude of the seasonality and hence assumes that the estimated contribution of El Niño to the 2015–2016 annual increment is representative of the contribution to the September 2016 concentration. A further assumption is that the annual increment is being added to a baseline that it not affected by the El Niño. With the Niño 3.4 anomaly already being strongly positive by September 2015, and the effects on land–atmosphere carbon fluxes already being evident in model simulations for that month (figure 5), this assumption may not be entirely valid. However, an accurate assessment of the importance of this goes beyond the limits of our simple methodology here, so provides scope for further research.

## 6. Conclusion

A record CO_2_ rise between 2015 and 2016 was successfully forecast in advance, using a seasonal forecast of sea surface temperatures and a statistical relationship with CO_2_. The successful calculation of the annual CO_2_ rise is robust to updates to the input data using observed SSTs and more recent data on emissions. The 2015/2016 CO_2_ rise was faster than the average over previous decade, due to the impact of El Niño acting mainly on tropical ecosystems. The annual CO_2_ rise had barely increased over the previous decade due to a prevalence of La Niña conditions, so the cessation of this influence also contributed to the large increase in CO_2_ rise in 2016. The 2015/2016 CO_2_ rise was also faster than that following the previous large El Niño in 1997/1998, due to the increase in anthropogenic emissions since then. 2016 was the first year in the Mauna Loa record when monthly CO_2_ concentrations remained above the symbolic threshold of 400 ppm all year, and previously, we had suggested that this would not have been the case without the influence of the El Niño. However, a revised estimate suggests that the annual minimum CO_2_ concentration at Mauna Loa may have remained above 400 ppm even in the absence of El Niño, due to ongoing anthropogenic emissions. The forecast of the CO_2_ annual increment has since become a regular product in the Met Office long-range forecast portfolio and a new component of the annual Global Carbon Budget. Further refinements to the forecast could be made, particularly concerning the shape of the seasonal cycle and the in-year CO_2_ growth rate.

## Supplementary Material

Monthly adjustment factors for forecast

## Supplementary Material

Published and corrected forecast monthly CO_2_

## Supplementary Material

Estimate of CO_2_ concentrations without El Nino

## Supplementary Material

Amplitude of seasonal cycle

## Supplementary Material

Revisions to emissions datasets

## Data accessibility

The Scripps Institute primary Mauna Loa CO_2_ record is available at http://scrippsco2.ucsd.edu/data/atmospheric_co2/primary_mlo_co2_record. The HadObs sea surface temperature data for the Niño 3.4 region are available at https://www.metoffice.gov.uk/hadobs/hadsst3/data/download.html. Code for the JULES model is available at http://jules-lsm.github.io/access_req/JULES_access.html. Code for producing the CO_2_ forecast from the SST forecast is available at https://github.com/dougmcneall/co2-forecast.
