# Supplementary material for: A successful prediction of the record CO2 rise associated with the 2015/2016 El Niño
Source: Philos Trans R Soc Lond B Biol Sci. 2018 Oct 8;373(1760):20170301. doi: 10.1098/rstb.2017.0301 (PMC6178439; doi:10.1098/rstb.2017.0301)
Supplement: Monthly adjustment factors for forecast [file rstb20170301supp1.pdf]

# A successful prediction of the record $CO_2$ rise associated with the 2015/16 El Niño

Richard A. Betts, Chris D. Jones, Jeff. R. Knight, Ralph. F. Keeling, John. J. Kennedy, Andrew J. Wiltshire, Robbie M. Andrew, Luiz E. O. C. Aragao

**Table S1.** Monthly adjustment factors. These are added to the forecast annual mean $CO_2$ concentration to give the forecast monthly values.

| **Month** | **Difference from annual mean (ppm)** |
|---|---|
| January | -0.83 |
| February | -0.06 |
| March | 0.79 |
| April | 2.25 |
| May | 3.12 |
| June | 2.27 |
| July | 0.65 |
| August | -1.35 |
| September | -2.97 |
| October | -2.83 |
| November | -1.26 |
| December | 0.20 |
