# Supplementary material for: A successful prediction of the record CO2 rise associated with the 2015/2016 El Niño
Source: Philos Trans R Soc Lond B Biol Sci. 2018 Oct 8;373(1760):20170301. doi: 10.1098/rstb.2017.0301 (PMC6178439; doi:10.1098/rstb.2017.0301)
Supplement: Published and corrected forecast monthly CO2 [file rstb20170301supp2.pdf]

A successful prediction of the record CO_2 rise associated with the 2015/16 El Niño

Richard A. Betts, Chris D. Jones, Jeff. R. Knight, Ralph. F. Keeling, John. J. Kennedy, Andrew J. Wiltshire, Robbie M. Andrew, Luiz E. O. C. Aragao

Table S2. Monthly mean CO_2 concentrations (ppm) for 2016 in published and corrected forecast, and observations.

Month	Published forecast	Corrected forecast	Observed
January	403.62	403.34	402.64
February	404.39	404.11	404.16
March	405.24	404.96	404.86
April	406.70	406.42	407.57
May	407.57	407.29	407.65
June	406.72	406.44	407.00
July	405.10	404.82	404.50
August	403.10	402.82	402.23
September	401.48	401.20	401.01
October	401.62	401.34	401.50
November	403.19	402.91	403.64
December	404.65	404.37	404.55
