# Supplementary material for: A successful prediction of the record CO2 rise associated with the 2015/2016 El Niño
Source: Philos Trans R Soc Lond B Biol Sci. 2018 Oct 8;373(1760):20170301. doi: 10.1098/rstb.2017.0301 (PMC6178439; doi:10.1098/rstb.2017.0301)
Supplement: Estimate of CO2 concentrations without El Nino [file rstb20170301supp3.pdf]

A successful prediction of the record CO_2 rise associated with the 2015/16 El Niño

Richard A. Betts, Chris D. Jones, Jeff. R. Knight, Ralph. F. Keeling, John. J. Kennedy, Andrew J. Wiltshire, Robbie M. Andrew, Luiz E. O. C. Aragao

Table S3. Hindcast monthly CO_2 concentrations (ppm) for 2016 with and without the effects of the El Niño SST anomaly, compared to observations. Two methods are used for the "no El Niño" case:

Column 3: addition of the previous decadal mean trend of 2.1 ppm yr^{-1} to the observed 2015 monthly concentrations (column 3)

Column 4: calculation of the 2016 annual mean CO_2 concentration using the regression, with a Niño3.4 SST anomaly of zero, then addition of the monthly adjustment factors (Table S1).

	**Hindcast**	**No El Niño (from trend)**	**No El Niño (from regression)**	**Observed**
Annual	404.10	402.99	403.31	404.28
Jan	403.27	401.95	402.48	402.64
Feb	404.04	402.41	403.25	404.16
Mar	404.89	403.61	404.10	404.86
Apr	406.35	405.55	405.56	407.57
May	407.22	406.20	406.43	407.65
Jun	406.37	404.98	405.58	407.00
Jul	404.75	403.71	403.96	404.50
Aug	402.75	401.10	401.96	402.23
Sep	401.13	399.60	400.34	401.01
Oct	401.27	400.38	400.48	401.50
Nov	402.84	402.34	402.05	403.64
Dec	404.30	403.99	403.51	404.55
