# Supplementary material for: A successful prediction of the record CO2 rise associated with the 2015/2016 El Niño
Source: Philos Trans R Soc Lond B Biol Sci. 2018 Oct 8;373(1760):20170301. doi: 10.1098/rstb.2017.0301 (PMC6178439; doi:10.1098/rstb.2017.0301)
Supplement: Amplitude of seasonal cycle [file rstb20170301supp4.pdf]

A successful prediction of the record CO_2 rise associated with the 2015/16 El Niño

Richard A. Betts, Chris D. Jones, Jeff. R. Knight, Ralph. F. Keeling, John. J. Kennedy, Andrew J. Wiltshire, Robbie M. Andrew, Luiz E. O. C. Aragao

Stationarity of amplitude of seasonal cycle

Our analysis assumes a stationary seasonal cycle which is added to the forecast increment in annual mean concentrations. The use of an additive cycle for maximum and minimum monthly concentrations is supported by further seasonality analysis demonstrating no trend in amplitude of an additive-derived seasonal cycle (Figure S2).

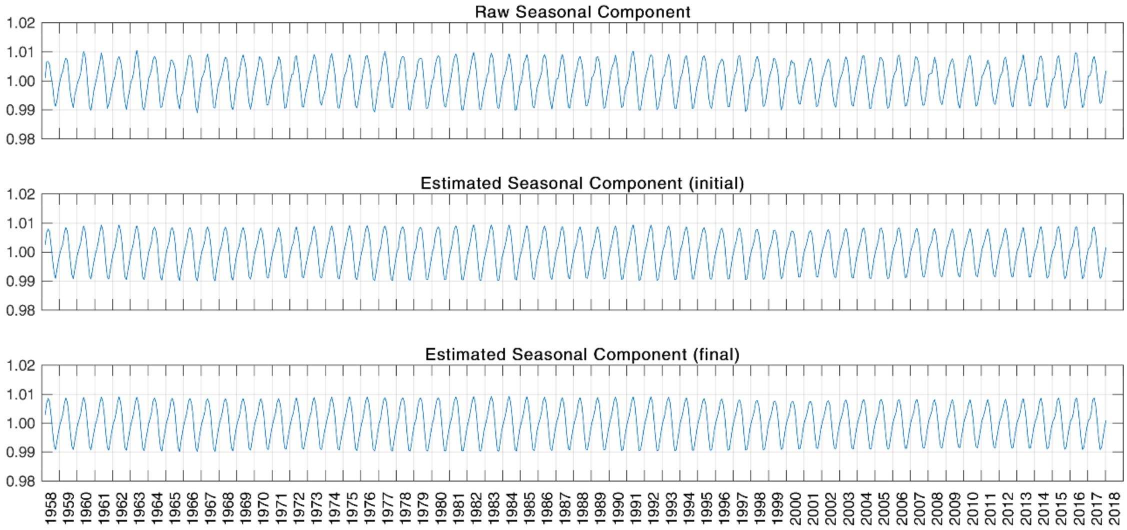

Figure S1. Seasonal cycle of Mauna Loa CO_2 concentrations.
