# Supplementary material for: A successful prediction of the record CO2 rise associated with the 2015/2016 El Niño
Source: Philos Trans R Soc Lond B Biol Sci. 2018 Oct 8;373(1760):20170301. doi: 10.1098/rstb.2017.0301 (PMC6178439; doi:10.1098/rstb.2017.0301)
Supplement: Revisions to emissions datasets [file rstb20170301supp5.pdf]

A successful prediction of the record CO_2 rise associated with the 2015/16 El Niño

Richard A. Betts, Chris D. Jones, Jeff. R. Knight, Ralph. F. Keeling, John. J. Kennedy, Andrew J. Wiltshire, Robbie M. Andrew, Luiz E. O. C. Aragao

Revision of emissions datasets

The annual Global Carbon Budget published by the Global Carbon Programme (GCP) includes updated datasets of historical emissions (Figure S1), which the addition of the most recent year of data and also a revision of previous years based on new information and methods.

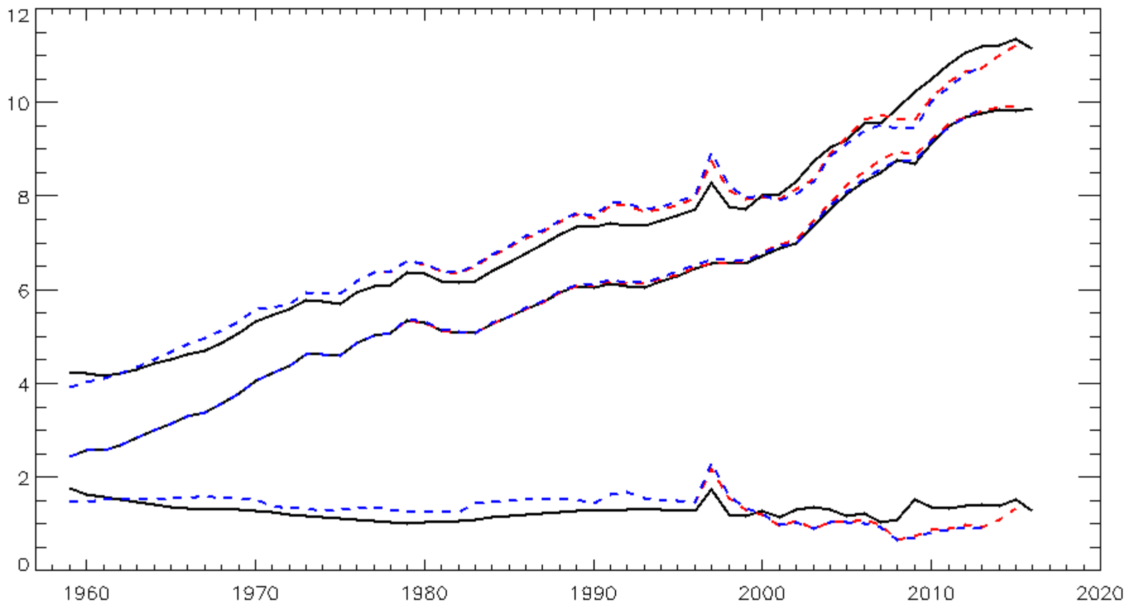

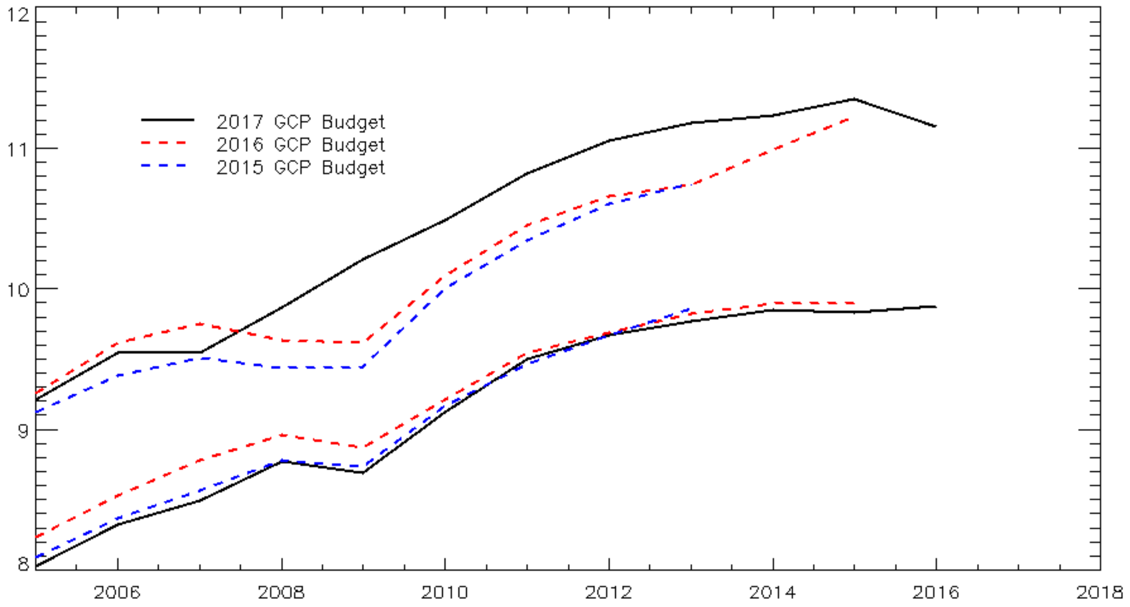

Figure S2. Timeseries of global mean annual emissions published by the Global Carbon Project (GCP) in 2015 (blue)[11], 2016 (red)[12] and 2017 (black)[13]. In each panel, the lower group of curves shows emissions from land use change, the middle group shows emissions from fossil fuel burning and cement production, and the top group shows the total emissions. The upper panel shows data from 1958, the start year of the Mauna Loa CO_2 record and hence the start of the period used to calculate the regression for the CO_2 forecast. The lower panel shows the most recent decade in order to allow more details to be seen.
